# Supplementary material for: Telehealth Exercise Training in Peripheral Arterial Disease (TEXTPAD) study: A pilot randomised controlled trial in socioeconomically disadvantaged populations
Source: PLoS One. 2025 Nov 4;20(11):e0327633. doi: 10.1371/journal.pone.0327633 (PMC12585064; doi:10.1371/journal.pone.0327633)
Supplement: Attachment — Submitted filename: PLOS ONE FEEDBACK.docx [file pone.0327633.s004.docx]

SUMMARY

This study aimed to assess feasibility and acceptability of a tele-health intervention, which constituted lifestyle modification targeted educative reading materials, videos and one-one interaction with specially trained health improvement practitioners. Participants attended a phone-based educative/counselling session once a week via a phone call and participated in a zoom-based exercise therapy twice a week. The control intervention constituted the usually offered lifestyle education and counselling aimed at increasing physical activity levels (e.g doing 30 minutes of exercise three times a week) and adoption of healthy behaviours, which are offered at the PAD clinic. A total of n=37 participants living with PAD were recruited, instead of an expected total of n=60 participants. An exploratory analytical approach was conducted to answer the study’s set objectives.

ABSTRACT

1. Authors conclude that the intervention is ‘feasible and acceptable’. In the study protocol, it is clearly indicated that qualitative interviews were (possibly) done to explore the experiences of participants after receiving the intervention, to determine the ‘acceptability’ of the program. Nevertheless, this aspect has not been reported in the study methods of this paper (measures, analytical plan) and results sections; yet it appears in the abstract’s conclusion. How did the authors draw this conclusion?

INTRODUCTION

1. Page 3: line 10. Authors should consider putting a space between the ‘quality of life’ and (9)
2. Page 3: Line 14. ‘and weight management (1). Authors should consider putting a space between ‘management and (1).
3. Pages 3-4: Authors describe the aim of the study, which are mainly focusing on assessing the feasibility and ‘acceptability’ of the telehealth intervention. However, the subsequent sections (including results section) have not narrated the assessment of acceptability aspect, and why it was important to assess. It may be important to insert such information to indicate the relevance of the approach/intervention.

METHODS

Inclusion and exclusion criteria

1. Authors indicate that they enrolled participants aged <40 years. Did this age limit contribute to the low turnover rate?
a. Why limiting the age to <40 years when in the introduction it is mentioned that PAD particularly affect those aged over 60 years?
2. Were the patients on secondary prevention medications? This has not been mentioned in the protocol either.
3. Authors have also reported that patients with ‘chronic limb-threatening ischemia’ were excluded. Was this based on a known existing diagnosis?

Intervention

1. Authors report that ‘prescribed exercise’ was done at home (intervention arm). It is not clear if the participants underwent baseline screening to establish their appropriate exercise target zone (for both safety and effectiveness of the intervention)?
2. Authors also mention that the intensity was prescribed and progressed between 12-14 Borg-RPE-20 scale. The intervention comprised of both aerobic and strength exercises, were the strength exercises prescribed? Considering that Bog-RPE scale is suitable for monitoring aerobic capacity. How did authors consider safety of the participants in using ‘household objects’ for strength training if no personalised prescription was done?
3. I am assuming that all participants had access to mobile phones (smart phones) to access all intervention materials? Or were they supported with such resources (the 8% and 15% with no device/internet access)?
4. Are the health improvement practitioners also trained in ‘exercise prescription’ or they are promoted to implement already prescribed exercise therapy? Is the dream to have a partially structured physical activity intervention in this case?

RESULTS AND DISCUSSION

1. On page 7, results section, authors indicate that there were n=99 eligible participants, yet the flowchart indicate n=60 as passing the pre-set inclusion criteria.
2. Due to the limited interpretive nature of the quantitative descriptive data, authors may consider presenting the qualitative data drawn from the qualitative individual interviews aimed at assessing the acceptability of the intervention. Hearing from the participants may be a better supplementary information, key to understanding the technicalities of the intervention.

GENERAL COMMENTS

1. Authors refer to study participants as ‘patients’. To reflect patients’ active role and contribution towards the research process (compared to their passiveness in clinical care ‘delivery-access’ processes) it is generally recommended to refer to them as ‘participants’ in research. Authors may wish to reconsider this.
